# Therapeutic targets for inflammatory bowel disease: proteome-wide Mendelian randomization and colocalization analyses

**DOI:** 10.1016/j.ebiom.2023.104494

**Published:** 2023-02-27

**Authors:** Jie Chen, Fengzhe Xu, Xixian Ruan, Jing Sun, Yao Zhang, Han Zhang, Jianhui Zhao, Jie Zheng, Susanna C. Larsson, Xiaoyan Wang, Xue Li, Shuai Yuan

**Affiliations:** a Department of Big Data in Health Science, School of Public Health and the Second Affiliated Hospital, Zhejiang University School of Medicine, Hangzhou, Zhejiang, China; b Key Laboratory of Growth Regulation and Translational Research of Zhejiang Province, School of Life Sciences, Westlake University, Hangzhou, China; c Department of Gastroenterology, The Third Xiangya Hospital, Central South University, Changsha, China; d Department of Gastroenterology, Ruijin Hospital, Shanghai Jiao Tong University School of Medicine, Shanghai, China; e Department of Endocrine and Metabolic Diseases, Shanghai Institute of Endocrine and Metabolic Diseases, Ruijin Hospital, Shanghai Jiao Tong University School of Medicine, Shanghai, China; f Unit of Cardiovascular and Nutritional Epidemiology, Institute of Environmental Medicine, Karolinska Institutet, Stockholm, Sweden; g Unit of Medical Epidemiology, Department of Surgical Sciences, Uppsala University, Uppsala, Sweden

**Keywords:** Inflammatory bowel disease, Mendelian randomization, Protein, Therapeutic target

## Abstract

**Background:**

Identifying new drug targets for inflammatory bowel disease (IBD) is urgently needed. The proteome is a major source of therapeutic targets. We conducted a proteome-wide Mendelian randomization (MR) and colocalization analyses to identify possible targets for IBD.

**Methods:**

Summary-level data of 4907 circulating protein levels were extracted from a large-scale protein quantitative trait loci study including 35,559 individuals. Genetic associations with IBD and its subtypes were obtained from the Inflammatory Bowel Disease Genetics Consortium (25,024 cases and 34,915 controls), the FinnGen study (7206 cases and 253,199 controls), and the UK Biobank study (7045 cases and 449,282 controls). MR analysis was conducted to estimate the associations between protein and IBD risk. The colocalization analysis was used to examine whether the identified proteins and IBD shared casual variants.

**Findings:**

Genetically predicted levels of 3, and 5 circulating proteins were associated with IBD and ulcerative colitis (UC), respectively. With high supporting evidence of colocalization, genetically predicted MST1 (macrophage stimulating 1) and HGFAC (hepatocyte growth factor activator) levels were inversely associated with IBD risks. The associations of STAT3 (signal transducer and activator of transcription 3), MST1, CXCL5 (C-X-C motif chemokine ligand 5), and ITPKA (inositol-trisphosphate 3-kinase A) with the risk of UC were supported by colocalization analysis.

**Interpretation:**

The proteome-wide MR investigation identified many proteins associated with the risk of IBD. MST1, HGFAC, STAT3, ITPKA, and CXCL5 deserve further investigation as potential therapeutic targets for IBD.

**Funding:**

SCL is supported by research grants from the 10.13039/501100006636Swedish Research Council for Health, Working Life and Welfare (Forte; grant no. 2018-00123) and the 10.13039/501100004359Swedish Research Council (Vetenskapsrådet; grant no. 2019-00977). XYW is supported by research grants from the 10.13039/501100001809National Natural Science Foundation of China (81970494) and 10.13039/100016104Key Project of Research and Development Plan of Hunan Province (2019SK2041). XL is supported by research grants from the Natural Science Fund for Distinguished Young Scholars of Zhejiang Province (LR22H260001).

**Research in context**

**Evidence before this study**

The benefits of current drug therapies for inflammatory bowel disease (IBD) including Crohn's disease (CD) and ulcerative colitis (UC) are far from optimal. Combining a proteomic approach with Mendelian randomization (MR) might constitute attractive targets for potential therapeutics.

**Added value of this study**

In the current study, we conducted a proteome-wide MR and colocalization analyses to capture the potential causal associations of over 4000 circulating proteins with IBD and its subtype. By triangulation of two analyses, we found evidence that genetically predicted higher levels of MST1 (macrophage stimulating 1) and HGFAC (hepatocyte growth factor activator) were associated with decreased IBD risks. Genetically predicted higher levels of circulating STAT3 (signal transducer and activator of transcription 3) were associated with an increased risk UC, whereas genetically predicted higher levels of circulating MST1, CXCL5 (C-X-C motif chemokine ligand 5), and ITPKA (inositol-trisphosphate 3-kinase A) was associated with a reduced risk of UC.

**Implications of all the available evidence**

The current study suggests that MST1, HGFAC, STAT3, ITPKA, and CXCL5 may be prioritized as potential drug targets for IBD or UC. These findings need further investigation in basic and clinical studies.

## Introduction

Inflammatory bowel disease (IBD) is a group of refractory chronic inflammatory diseases in the intestine, including Crohn's disease (CD) and ulcerative colitis (UC), causing a significant caregiving burden for individuals, families, and society.^1^ Conventional IBD therapies are not always effective^2^ and its complex biological aetiology limits the development of new drugs. Circulating proteins as key regulators of molecular pathways are always treated as targets of pharmacal therapies. Previous studies have reported that circulating proteins may involve in the development of IBD and have therapeutic potentials.^3^^,^^4^ A nested case–control study of 200 CD patients found that proteins of the complement cascade and lysosomes, innate immune response were associated with CD.^4^ A cohort study including 72 UC patients found that stromelysin-2, C-X-C motif chemokine ligand 9, signalling lymphocytic activation molecule, C-X-C motif chemokine 11, and monocyte chemotactic protein 1 were upregulated prior to UC diagnosis.^5^ However, the observed associations of circulating proteins with IBD were obtained from observational studies that may be susceptible to confounding and reverse causation. Additionally, randomized control trials are not feasible to explore the causal associations of thousands of proteins with IBD, let alone without conclusive evidence.

Mendelian randomization (MR) employed genetic variants as an instrumental variable for the exposure (e.g., a circulating protein) to reinforce causal inference. MR is less prone to confounding because genetic variants are randomly assorted at conception and thus not related to environmental and self-adopted factors. A previous MR analysis including 464 CD patients and 484 UC patients examined the causal associations between 1300 serum metabolites and IBD.^3^ Recent studies on genetic associations with circulating protein levels in a large sample provide opportunities to investigate the causal effects of circulating proteins on IBD in a comprehensive way. Here, we performed a proteome-wide MR analysis plus colocalization analysis for IBD and its subtypes (CD and UC) to explore potential therapeutic targets for the disease.

## Methods

### Study design and ethics

**Fig. 1:**
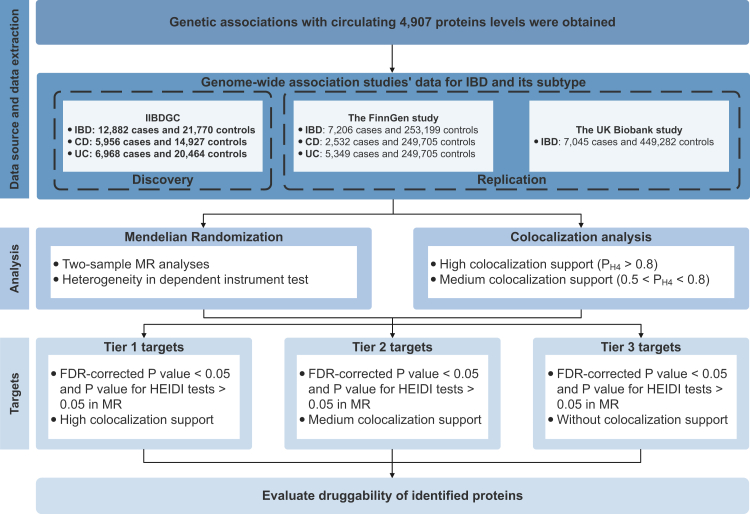
**Study design.** IIBDGC, the International Inflammatory Bowel Disease Genetics Consortium; IBD, inflammatory bowel disease; CD, Crohn's disease; UC, ulcerative colitis; FDR, false discovery rate; HEIDI, heterogeneity in dependent instrument; MR, Mendelian Randomization.

The study design is presented in Fig. 1. The study is based on publicly available data from a large-scale genome-wide association study on blood proteome (https://www.decode.com/summarydata/),^6^ the International Inflammatory Bowel Disease Genetics Consortium (IIBDGC, https://www.ibdgenetics.org/),^7^ the FinnGen study (https://www.finngen.fi/en),^8^ and the UK biobank study (https://cnsgenomics.com/content/data)^9^ (Supplementary Table S1). Included studies had been approved by corresponding ethical review committees.

### Proteomic data source

Summary-level statistics of genetic associations with levels of 4907 circulating proteins were extracted from a large-scale protein quantitative trait loci (pQTL) study in 35,559 Icelanders.^6^ Proteomic profiling was performed by a multiplexed, modified aptamer-based binding assay (SOMAscan version 4). The levels of protein were rank-inverse normal transformed by age and sex. The residuals were standardized using rank-inverse normal transformation and the standardized values were treated as phenotypes in the genome-wide association analyses under the BOLT-LMM linear mixed model. Details on the GWAS can be found in the original publication.^6^ The current study included the proteins with pQTLs available at the genome-wide significance level (*P* < 5 × 10^−8^) in two-sample MR. All proteins with summary-level data were included in the colocalization analysis.

### Outcome data sources

Summary-level data for IBD and its subtypes (CD and UC) were available in IIBDGC, the FinnGen study, and the UK Biobank study. IIBDGC comprised 12,882 IBD cases, 5956 CD cases, and 6968 UC cases. All participants of IIBDGC were of European ancestry.^7^ Cases were diagnosed by accepted radiologic, endoscopic, and histopathologic evaluations in IIBDGC. The genetic associations were adjusted for age, sex, and up to 20 genetic principal components. We also obtain summary-level data on IBD and its subtype from the R6 data release of the FinnGen study.^8^ Cases were defined by International Classification of Disease (ICD) codes (ICD-8 (5630), ICD-9 (555), and ICD-10 (K50) for 2532 CD cases; ICD-8 (5631, 569), ICD-9 (556), ICD-10 (K51) for 5349 UC cases. IBD contained CD and UC and the ICD codes that were unspecified. The UK Biobank study is a large multicenter cohort study that recruited more than 500,000 European participants between ages 37 and 73 years across the United Kingdom. In this study, summary-level statistics of genetic associations with IBD were extracted from a GWAS conducted by Wu et al.^9^ IBD diagnoses were derived from records of death register, self-reported, hospital admission or primary care record, resulting in a total of 7045 cases and 449,282 controls. The IIBDGC GWASs were employed in the discovery stage, and the GWASs from the UK Biobank study and the FinnGen study were employed for replication. There is no overlapping among these 3 data sources.

### MR analysis

We performed two-sample MR analysis based on index SNPs for proteins to capture the associations between circulating proteins and the risk of IBD and its subtypes.^10^ The odds ratios (ORs) and corresponding confidence intervals (CIs) of the associations between proteins and the outcomes were calculated using the Wald ratio and the delta method, respectively. Summary-data-based MR test using multiple SNPs (multi-SNPs-SMR) instrumented for each circulating protein was employed as a sensitivity analysis which provided significance levels to strengthen the evidence from the primary analysis. The heterogeneity in dependent instrument (HEIDI) test was used to distinguish a pleiotropic model from a linkage model, compared with most other methods based on GWAS and molecular QTL data.^10^ The association with *P* value in HEIDI test <0.05 was considered likely caused by pleiotropy and thus removed from the further analyses. SMR analysis was performed using the SMR software tool (SMR v1.0.3).^10^ We used the false discovery rate (FDR) at ɑ = 0.05 based on Benjamini–Hochberg method for multiple testing.

### Colocalization analysis

We conducted colocalization analysis using the coloc R package^11^ to test whether identified associations between proteins and IBD and subtypes were driven by linkage disequilibrium. For each locus, the Bayesian method assessed the support for the following five exclusive hypotheses: 1) no association with either trait; 2) association with trait 1 only; 3) association with trait 2 only; 4) both traits are associated, but distinct causal variants were for two traits; and 5) both traits are associated, and the same shares causal variant for both traits.^12^ The analysis provides posterior probabilities for each hypothesis testing (H0, H1, H2, H3, and H4). We set prior probabilities of the SNP being associated with trait 1 only (p1) at 1 × 10^−4^; the probability of the SNP being associated with trait 2 only (p2) at 1 × 10^−4^; and the probability of the SNP being associated with both traits (p12) at 1 × 10^−5^ two signals were considered to have strong evidence of colocalization if the posterior probability for shared causal variants (*P*_*H4*_) was ≥0.8. Medium colocalization indication was defined as 0.5 < *P*_*H4*_ < 0.8. For those proteins that were not available in MR (i.e., due to *P* value from pQTL >5 × 10^−8^), the colocalization analysis can still offer insights into understanding the relationship between genetic signals at a locus of circulating proteins and IBD.

Protein-outcome associations with FDR-corrected *P* value < 0.05 and *P* value for HEIDI tests >0.05 in MR were subsequently classified into three groups. Proteins with high-support evidence of colocalization (*P*_H4_> 0.8) were considered tier 1 targets; proteins with medium-support evidence of colocalization (0.5 < *P*_H4_ < 0.8) were considered tier 2 targets; the remaining proteins were considered tier 3 targets.

### Druggable proteins identification

To assess the druggability of identified proteins, we searched identified proteins in DrugBank, Dependency Map, the Connectivity Map, the ChEMBL databases, and in a list of druggable genes from a previous study.^13^ For proteins identified in drug databases, information on the drug name and the process of drug development was documented. To assess the potential druggability, we classified these proteins into four categories: 1) approved (one or more drugs targeting the specific protein have been approved); 2) in clinical trials (targeting drugs are currently studied in clinical trials); 3) preclinical (targeting drugs are in preclinical pipelines); 4) druggable (proteins could not be identified in drug database but listed as druggable targets).

### Role of funders

The funding sources had no role in the design of this study and did not have any role in data collection, data analyses, interpretation, writing of report, or decision to submit results.

## Results

### Proteome-wide MR analysis

**Fig. 2:**
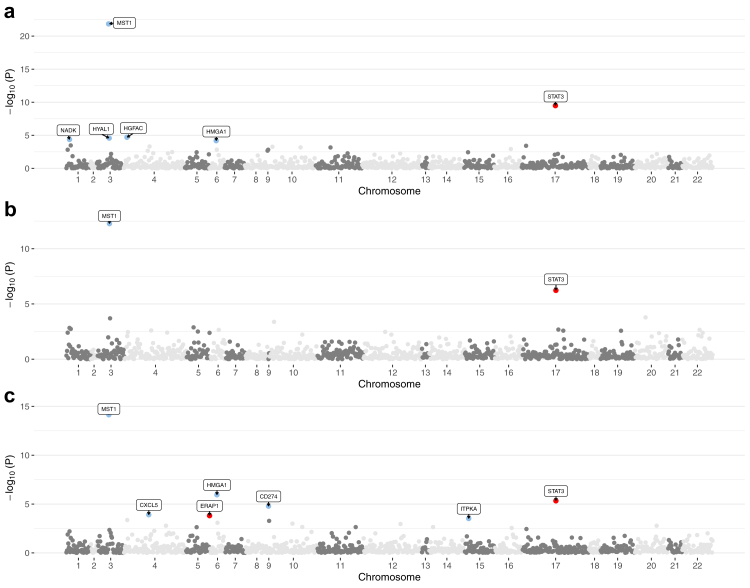
**Manhattan plots for associations of genetically predicted 1110 circulating proteins levels with IBD and its subtype in MR analysis**. a) Associations of genetically predicted circulating protein levels with IBD; b) associations of genetically predicted circulating protein levels with CD; c) associations of genetically predicted circulating protein levels with UC. Labelled and colour genes refer to MR findings with FDR-corrected *P* < 0.05 (two-sample MR analysis). Red genes indicate the positive effect of the circulating proteins on outcomes; blue genes indicate the negative effect of the circulating proteins on outcomes. Results are plotted by gene start position.

The study examined the MR associations between 1110 proteins with available index pQTL signals and the risk of IBD outcomes. The overview of results of MR analysis is presented in Fig. 2. We identified 93 protein-IBD pairs at the marginal significance level (*P* < 0.05 for two-sample MR analysis). Eighteen of the associations did not survive in HEIDI test due to possible pleiotropy (Supplementary Table S2). After removing associations that did not survive HEIDI test and multiple testing correction, genetically predicted higher levels of 3 circulating proteins including MST1 (macrophage-stimulating protein), NADK (Nicotinamide adenine dinucleotide kinase), HGFAC (Hepatocyte growth factor activator) were significantly associated with a decreased risk of IBD (Fig. 2 and Supplementary Table S2). For 1-SD increment of genetically predicted levels of protein, the odds ratio (ORs) of IBD was 0.86 (95% CI 0.84–0.89) for MST1, 0.54 (95% CI 0.40–0.73) for NADK, and 0.92 (95% CI 0.89, 0.96) for HGFAC. The results for NADK remained stable in SMR analysis (Supplementary Table S2). The associations for MST1 and NADK were replicated in the UK Biobank and the FinnGen study, and the association for HGFAC was directionally consistent in replication analysis (Supplementary Table S3).

After the removal of the associations that did not survive in the HEIDI test, there were 71 proteins associated with CD at *P* < 0.05 (two-sample MR analysis) (Supplementary Table S4). However, none of the associations survived after the Benjamini-Hochberg adjustment (Supplementary Table S4). Likewise, 67 nominal UC-specific associations were identified (Fig. 2 and Supplementary Table S5). After correction for multiple testing, genetically predicted higher levels of circulating STAT3 (signal transducer and activator of transcription 3) levels were associated with an increased risk of UC, while genetically predicted higher levels of circulating proteins including MST1, CD274 (cluster of differentiation 274), CXCL5 (C-X-C motif chemokine 5) and ITPKA (inositol-trisphosphate 3-kinase A) were associated with a decreased risk of UC. Per SD increment of genetically predicted levels of protein, the OR of UC was 0.86 (95% CI 0.83–0.90) for MST1, 1.99 (95% CI 1.48–2.68) for STAT3, 0.65 (95% CI 0.53–0.79) for CD274, 0.50 (95% CI 0.35–0.71) for CXCL5, and 0.28 (95% CI 0.14–0.56) for ITPKA. In the sensitivity analysis using multi-SNPs-SMR, the associations for STAT3, CD274, CXCL5, and ITPKA remained (Supplementary Table S5). The associations of MST1 and STAT3 with the risk of UC were replicated in the FinnGen study (Supplementary Table S6). MST1 was the only overlapping protein between IBD and UC and the associations of MST1 with IBD and UC were in the same direction.

### Colocalization analysis

**Fig. 3:**
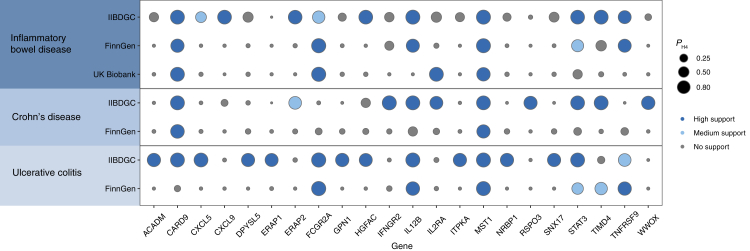
**High support evidence for colocalization between circulating proteins and outcomes in discovery and replication datasets**. Circle size indicates the colocalization *P* value for H4 (colocalization analysis) and the colour of the circle indicate the classification of the evidence.

We conducted colocalization analyses of circulating proteins with IBD and its subtype outcomes. We first tested whether the identified associations of the circulating protein with IBD and its subtypes shared causal variants in IIBDGC (Table 1). The high support of colocalization evidence was observed between 2 proteins (MST1 and HGFAC) and IBD, which were identified as tier 1 (Table 1 and Fig. 3). In addition, four proteins including MST1, CXCL5, ITPKA, and STAT3 were colocalized with UC associations with high support of evidence (Table 1 and Fig. 3), which were also identified as tier 1. MST1 was replicated in the FinnGen study or the UK Biobank study for IBD and UC with high support evidence (Fig. 3). The remaining proteins–outcome pairs with limited evidence of colocalization were ascertained as tier 3 targets.

**Table 1:** Mendelian randomization analysis and colocalization of circulating proteins with inflammatory bowel disease and its subtypes in IIBDGC.

Outcomes	Proteins	Mendelian randomization				Colocalization Analysis *P*_*H4*_	Targets
		OR (95% CI)^a^	*P* value	*P* value after FDR adjustment	*P* value for HEIDI test		
IBD	MST1	0.86 (0.84, 0.89)	1.41E-22	1.52E-19	0.179	0.980	Tier 1 targets
	HGFAC	0.92 (0.89, 0.96)	1.99E-05	7.14E-03	0.263	0.934	Tier 1 targets
	NADK	0.54 (0.40, 0.73)	4.20E-05	9.06E-03	0.372	0.391	Tier 3 targets
UC	STAT3	1.99 (1.48, 2.68)	4.63E-06	0.002	0.059	0.954	Tier 1 targets
	MST1	0.86 (0.83, 0.90)	7.16E-15	7.71E-12	0.146	0.982	Tier 1 targets
	ITPKA	0.28 (0.14, 0.56)	3.00E-04	4.61E-02	0.660	0.853	Tier 1 targets
	CXCL5	0.50 (0.35, 0.71)	1.23E-04	2.66E-02	0.526	0.940	Tier 1 targets
	CD274	0.65 (0.53, 0.79)	1.65E-05	0.004	0.138	7.34E-07	Tier 3 targets

CI, confidence interval; FDR, false discovery rate; OR, odds ratio.

a The OR of the outcome was scaled to one standard deviation increase in genetically predicted circulating protein levels.

We also performed colocalization analysis for proteins without pQTL at the genome-wide significance level. Circulating proteins with high support evidence of colocalization are presented in Fig. 3. In detail, IBD had high support for colocalization with 9 proteins including CARD9 (caspase recruitment domain-containing protein 9), CXCL9, ERAP2 (endoplasmic reticulum aminopeptidase 2), HGFAC, IL12B (interleukin-12 subunit beta), MST1 (hepatocyte growth factor-like protein), STAT3, TIMD4 (t-cell immunoglobulin and mucin domain-containing protein 4), and TNFRSF9 (tumour necrosis factor receptor superfamily member 9); of these, CARD9, IL12B, MST1, and TNFRSF9 were validated in the replication dataset (Fig. 3 and Supplementary Table S7). For CD, we found 9 circulating proteins with high support colocalization evidence including CARD9, IFNGR2 (interferon gamma receptor 2), IL12B, IL2RA (interleukin-2 receptor subunit alpha), MST1, STAT3, TIMD4, and WWOX (WW domain-containing oxidoreductase) (Fig. 3). CARD9 and MST1 were replicated in the FinnGen study (Supplementary Table S8). UC had high support for colocalization with 14 circulating proteins including ACADM (medium-chain specific acyl-CoA dehydrogenase, mitochondrial), CARD9, CXCL5, DPYSL5 (dihydropyrimidinase-related protein 5), ERAP2, FCGR2A (low-affinity immunoglobulin gamma Fc region receptor II-a), GPN1 (GPN-loop GTPase 1), HGFAC, IL12B, ITPKA, MST1, NRBP1 (nuclear-receptor-binding protein), SNX17 (sorting nexin-17), and STAT3 (Fig. 3); FCGR2A, IL12B, and MST1 were replicated in the FinnGen study (Supplementary Table S9).

### Druggability of identified proteins

We searched seven circulating proteins identified in MR analysis as possible drug targets in drug databases. None of them were found to be drug targets for IBD (Supplementary Table S10). Drugs targeting MST1, ITPKA, CD274 were mainly designed to treat cancers. Several drugs targeting STAT3 were also identified, one of which has been approved to be used in tapeworm treatment. No drug information was found for HGFAC, NADK, or CXCL5. We also searched drug information on proteins with high support evidence of colocalization (Supplementary Table S10). One drug targeting IL12B named Ustekinumab has been approved to treat autoimmune diseases including IBD. Drugs targeting TNFRSF9, IL2RA, FCGR2A, IFNGR2 were found to be potential targets for cancers. No information was available for NADK, CARD9, ERAP2, TIMD4, WWOX, DPYSL5, GPN1 and SNX17 were in searched drug databases.

## Discussion

We conducted a proteome-wide MR and colocalization analyses to explore the causal roles of over 4000 circulating proteins in IBD and its subtype to provide preclinical clues for the drug development. MR analysis identified 3 protein-IBD associations and 5 protein-UC associations. After teasing out the possible effects of linkage disequilibrium by colocalization analysis, we found strong evidence that genetically predicted higher levels of MST1 and HGFAC was inversely associated with IBD risks. We also found that genetically predicted higher levels of circulating STAT3 were associated with an increased risk UC, whereas genetically predicted higher levels of circulating MST1, CXCL5, and ITPKA was associated with a reduced risk of UC.

In the current study, we identified MST1 as a potential target for both IBD and UC with robust evidence. MST1, one member of class II germinal centre kinases, was reported to function in mediating intracellular signalling induced by various stimuli. Previous studies have indicated that the MST1 gene was a risk locus for IBD^14^^,^^15^; furthermore, our study confirmed the association of MST1 with IBD and UC was causal. Interestingly, evidence from the experiment showed that MST1 was a negative regulator of TNF (tumour necrosis factor)-induced inflammatory signaling.^16^ Considering that TNF has been recognized as triggering inflammation and being involved in the pathogenesis of IBD, together with our findings on the casual associations of MST1 with IBD and UC, it is valuable to investigate the pathological role of MST1. Unlike MST1, the association between HGFAC and IBD was less explored. HGFAC converts hepatocyte growth factor to an active form in response to tissue injury.^17^ Animal experiments showed that HGFAC knockout mice are more susceptible to dextran sulfate sodium-induced colitis compared to wild-type mice.^18^^,^^19^

STAT3 has been known as a mediator for gene expression induced by many important cytokines including IL-6 (interleukin 6)^20^ and IL-23 (interleukin 23).^21^ Previous in vivo experiments showed that STAT3 was upregulated in intestinal tissue and circulation in active CD and UC patients.^22^^,^^23^ A meta-analysis found that the *STAT3* rs744166 polymorphism was associated with CD and UC susceptibility.^24^ With replication in the FinnGen study, our findings provided consistent evidence that genetically predicted circulating STAT3 levels were positively associated with the risk of UC; the HEIDI test and the colocalization analysis further ruled out the possibility of horizontal pleiotropy. Considering the central role of STAT3 in Th17 differentiation,^25^ this finding is particularly significant. However, the association between STAT3 and CD was not observed in the current study. The discrepancy is likely caused by reverse causation that the inflammatory responses increase the expression of STAT3 after the onset of the disease. Another possible explanation may be that the over-conservative HEIDI test threshold (*P* > 0.05)^10^ rejected the association between circulating STAT3 and CD and the high-support evidence of colocalization in the present study suggested the existence of the causal relationship. Our findings indicated that STAT3 inhibitors might have promising therapeutic potential as drug candidates. Mechanistically, it has been proved that IL-6 and IL-23 regulate the proliferation of Th17 cells through the Janus kinase (JAK)/STAT3 signalling pathway, which mediates autoimmune-mediated inflammation.^26^ The inhibitor of JAK which is the upstream protein of STAT3 has been demonstrated to benefit moderate-to-severe UC.^27^ Besides, clinical trials have also demonstrated the efficacy of Ustekinumab targeting IL-23 for UC.^28^^,^^29^ Studies have found that IL23/Th17 signalling pathway plays an essential role in the pathogenesis of UC. STAT3 is an important downstream member of this pathway. Further studies are needed to give mechanistic insight into how STAT3 deranges cytokine regulation and promotes the breakdown of intestinal homeostasis. In addition, whether STAT3 could serve as a potential therapeutic target deserves clinical trials.

ITPKA regulates actin dynamics^30^ and inactivated inositol triphosphate-dependent calcium release from the astrocyte endoplasmic reticulum.^31^ ITPKA is highly expressed in the brain, duodenum, small intestine, colon, and stomach.^32^ Although there was substantial evidence of ITPKA on carcinogenesis and metastasis,^33^ the role and regulation of ITPKA in inflammatory or immune disease remains less unknown. Based on integrating IBD GWAS with eQTLs from 203 anti-tumour necrosis factor-resistant CD patients, a previous study found that polymorphism rs28374715 was associated with decreased risk of IBD through downregulated ITPKA in blood and intestine.^34^ Consistent with previous findings, the study further provided evidence that genetically predicted ITPKA level was associated with a lower risk of UC but not CD. Based on our findings, genetically predicted ITPKA was inversely associated with risks of UC. This association was unlikely to be biased by pleiotropy, which was supported by the HEIDI test and colocalization analysis. Albeit the contribution of this protective mechanism in UC remains to be investigated, possible mechanisms might be implicated. ITP3K proteins phosphorylate the inositol 1,4,5-trisphosphate to inositol 1,3,4,5-trisphosphate,^35^ and the latter negatively regulates phosphatidylinositol-3,4,5- trisphosphate signalling in neutrophils which are associated with inflammation.^36^ We also provided evidence that genetically predicted CXCL5 levels were inversely associated with UC risk; however, literature on this association was scarce. CXCL5 belongs to the CXC chemokine family which is preferentially expressed in intestinal epithelium in IBD.^37^^,^^38^ These newly identified associations are worthy of further studies.

We also observed four proteins (FCGR2A, CARD9, IL12B, and TNFRSF9) with high-supporting evidence of colocalization. However, we could not rule out the possibility that these associations were caused by pleiotropy since these associations could not be examined in SMR which requires multiple genetic instruments. Thus, the associations for corresponding proteins need verification.

A strength of this investigation is that we employed MR and colocalization analyses that exploit genetic variants to estimate the causal effects of circulating proteins on IBD. MR design minimized bias due to confounding and reverse causality and thus improved the causal inference. Colocalization analysis has been proven a powerful tool in revealing the pleiotropic effects of certain loci on multiple traits. In addition, we used GWASs with large sample sizes, which increased the power to detect mild-to-moderate associations. We also performed the analyses in several datasets and the consistency of the results strengthened our findings. Another strength is that we confined our analysis to individuals of European ancestry, which minimized the population stratification bias.

Some limitations of our analysis are worth noting. Even though colocalization analysis ruled out possible bias caused by linkage disequilibrium, horizontal pleiotropy could not be minimized. Nevertheless, horizontal pleiotropy might be reduced by the HEIDI test with a conservative *P*-value threshold. Again, the population confinement minimizes the population structure bias; however, it limited the generalization of our findings to other populations. An additional limitation is that the interpretation of the posterior probabilities (*P*_*H4*_) in colocalization requires caution, which means a low *P*_*H4*_ may not indicate evidence against colocalization evidence in situations where *P*_*H3*_ is also low due to insufficient power. Moreover, our investigation focused on the proteins that were available in MR (only 1110 proteins with available index pQTL signals at the genome-wide significance threshold), and therefore it is likely to neglect the viable therapeutic targets. However, we conducted a comprehensive colocalization of circulating proteins to capture the potential candidate genes which may have a causal effect on IBD. Finally, the plasma proteome data were obtained from a cohort of Icelanders. However, data on IBD and its subtypes were mainly based on European populations. Differences in ancestries of data sources may introduce population structure bias even though top genetic principal components, an indicator of population structure, were adjusted for in the GWASs.

In summary, this study identified many associations of circulating proteins with the risk of IBS and its subtypes using an integrated genetic approach. By integrating data from drug databases, MST1, HGFAC, STAT3, ITPKA, and CXCL5 were prioritized as potential drug targets for IBD or UC, which need to be verified in future trials.

## Contributors

F.X and S.Y. had full access to all the data in the study and take responsibility for the integrity of the data and the accuracy of the data analysis. J.C., F.X., X.W., X.L., and S.Y. conceived and designed the study. F.X. and X.R. undertook the statistical analyses. J.C., X.R., and S.Y. wrote the first draft of the manuscript. J.C., F.X. and S.Y. are the study guarantor. J.C., F.X., X.R., J.S., Y.Z., H.Z., J.Z., J.Z., S.C.L., X.W., X.L., and S.Y. interpreted data, reviewed the paper, and made critical revision of the manuscript for important intellectual content. All authors read and approved the final version of the manuscript.

## Data sharing statement

All data analysed in this study can be obtained by a reasonable request to corresponding authors.

## Declaration of interests

All authors have completed the ICMJE uniform disclosure form at www.icmje.org/coi_disclosure.pdf and declare no competing interests.
